# BI-RADS application for breast cancer screening in primary healthcare settings: assessing protocol adherence and diagnostic validity

**DOI:** 10.3389/fonc.2025.1599759

**Published:** 2025-10-16

**Authors:** Xiang Li, Hong Wang, Hui-Fang Xu, Shao-Kai Zhang, Bing-Jie Zheng, Hai-Liang Li

**Affiliations:** ^1^ Department of Radiology, Affiliated Cancer Hospital of Zhengzhou University & Henan Cancer Hospital, Zhengzhou, China; ^2^ Department of Cancer Epidemiology, Affiliated Cancer Hospital of Zhengzhou University & Henan Cancer Hospital, Zhengzhou, China

**Keywords:** BI-RADS, breast cancer screening, primary healthcare setting, automated breast ultrasound, handheld ultrasound, mammography

## Abstract

**Background:**

The application performance of the Breast Imaging-Reporting and Data System (BI-RADS) in primary healthcare settings remains uncertain. The normativity of BI-RADS classification and the efficacy of breast cancer detection guided by BI-RADS classification were evaluated here.

**Methods:**

All data used in the current study were derived from a breast cancer screening cohort baseline database, which consists of 8,996 women aged 35–64 years from Central China. Participants aged 35–44 underwent automated breast ultrasound (ABUS) and handheld ultrasound (HHUS), while those aged 45–64 were screened with ABUS, HHUS, and mammography (MG). All imaging diagnoses were made by radiologists according to the BI-RADS 5^th^ edition classification system published by the ACR in 2013. The distribution of malignant imaging findings and inter-modality agreement on BI-RADS classifications were assessed. Based on pathological results, the area under the receiver operating characteristic (ROC) curve (AUC) was used to assess the performance of breast cancer screening according to BI-RADS-guided referrals.

**Results:**

Of individuals found with irregular morphology, 44%, 45.7% and 77.4% were classified as BI-RADS category 4 or higher for HHUS, ABUS and MG, respectively; For those with indistinct margins, the proportion was 81%, 77.5% and 40.8%, correspondingly; For grouped calcifications, they were 100% for HHUS and 85.7% for MG; Meanwhile, 72.7% and 88.9% not parallel (taller than wide) masses were categorized as BI-RADS category 4 for HHUS and ABUS. The concordance of BI-RADS classification was as high as 98.2% between HHUS and ABUS (Kappa = 0.726), whereas it was about 96% between ultrasound and MG (Kappa ranged from 0.21 to 0.25). The BI-RADS guided screening performance for breast cancer showed AUC values of 0.9935 for ABUS, 0.9529 for HHUS, 0.8983 for MG. If the BI-RADS diagnosis of MG was considered in ultrasound-negative women, only the HHUS’s performance was boosted, achieving an AUC of 0.9920.

**Conclusions:**

Radiologists at primary hospitals can effectively apply BI-RADS based on the malignant features they found. BI-RADS can provide a reliable framework for guiding breast cancer screening in primary healthcare settings.

## Background

The goal of breast cancer screening is to detect cancer at an early stage, thereby improving treatment outcomes and survival rates (1–3). The imaging modalities usually used in breast cancer screening include handheld ultrasound (HHUS), and mammography (MG). HHUS is widely used in primary healthcare settings due to its ease of operation, low cost, and effectiveness in detecting breast masses and cystic lesions (4). Technological advancements have led to the incorporation of automated breast ultrasound (ABUS) in breast cancer screening, providing higher resolution and precise spatial localization by volume imaging for clearer breast imaging and improved mass detection rates (5, 6). MG, which utilizes low-dose X-rays, is vital for early breast cancer detection, especially in women with less dense breast glandular tissue (7, 8).

However, imaging diagnostic accuracy is modulated by radiologists experience (9). To enhance diagnostic accuracy, the Breast Imaging-Reporting and Data System (BI-RADS) is a widely adopted classification system for interpreting breast imaging findings (10, 11). BI-RADS integrates imaging features, risk assessment, and clinical information to inform diagnostic and treatment decisions (12, 13). The BI-RADS classification ranges from 0 to 6, with BI-RADS category 4 and 5 indicating a higher likelihood of malignancy (14). BI-RADS was built on previous work focused on the positive predictive value of imaging features, by clarifying previous terms with an aim toward risk stratification. The malignancy indicators emphasized within BI-RADS encompass grouped or linear calcifications (15–17) and mass-related signs such as irregular shape, indistinct margin, not parallel (taller than wide) (18–21), along with other accompanying signs such as architectural distortion (22, 23), asymmetry (24, 25), and harder textures in tumors through ultrasound elastography (26, 27).

Despite advancements in imaging technology, the variance in clinical expertise, imaging equipment, and training levels across different levels of hospitals persists, leading to an uncertain efficacy of identifying and interpreting malignant signs and radiological diagnostic capabilities in breast cancer screening within the primary healthcare setting (12, 28). Therefore, this study aims to assess the application of BI-RADS classification in primary hospitals by evaluating the relationship between imaging features, BI-RADS classification outcomes, and pathological findings in breast cancer screening.

## Materials and methods

### Study design and population

Data used in the current study were extracted from a breast cancer screening cohort which consists of 8,996 women aged 35–64 years from Central China. BI-RADS diagnoses, pathological results and imaging characteristics were retrospectively analyzed. Participants enrolled in this screening cohort were local general women aged 35–64 years. The exclusion criteria included being pregnant, lactating, or planning to become pregnancy; had a history of breast tumor resection, contralateral breast surgery, breast augmentation, or percutaneous biopsy within the past 12 months; had a prior tumor diagnosis or treatment within the last 12 months; or exhibited suspicious signs without an imaging indication. This study was approved by the independent ethic committee of Henan Cancer Hospital (Approval Number: 19/109-1893).

### Imaging screening

In the current study, breast cancer imaging screening modalities included HHUS, ABUS and MG. Based on current screening guidelines (29, 30), which recommended MG for women aged ≥45 years, participants were stratified into two age groups: 35–44 years (screened with ABUS and HHUS) and 45–64 years (screened with ABUS, HHUS and MG).

HHUS was performed using the EADN U50 ultrasound device (frequency range: 7.0-16.0 MHz; EADN, Shenzhen, China), with detailed documentation of breast lesion characteristics. ABUS was operated using the SIUI IBUS BE3 (frequency: 5–12 MHz; Shantou Institute of Ultrasonic Instruments, Shantou, China) and Invenia ABUS (C15-6XW Reverse Curve™, frequency: 6–15 MHz; GE Healthcare, Hatfield, UK) devices for scanning. MG was conducted using the Hologic Selenia Dimensions system (Hologic, Massachusetts, USA). Ultrasound and MG images were interpreted by radiologists according to the BI-RADS 5^th^ edition classification system published by the ACR in 2013.

The key imaging findings serving as the basis for the BI-RADS classification were collected as follows: the size, shape, margin, orientation of the lesion, the presence of calcifications, and other associated signs of suspicious lesions (including architectural distortion, skin thickening, and hardness in elasticity-imaging) for HHUS and ABUS; The size, shape, and density of lesion, the presence of calcifications, and other associated signs (including architectural distortion, skin thickening, and the presence of asymmetries) for MG. Mass shape was categorized as regular(including round, oval) or irregular; Orientation was classified as parallel (long axis of lesion is parallel to the skin, also referred to as wider than tall) or not parallel (long axis of lesion is not parallel to the skin, also referred to as taller than wide); The margin was described as circumscribed or indistinct; Calcifications were classified as grouped, linear or benign-appearing. Architectural distortion was defined as a localized distorted breast parenchyma with no definite mass visible; asymmetries was defined as an asymmetric dense shadow of fibro glandular tissue without a clear three-dimensional outline or distinct margin when compared with the corresponding location on the contralateral breast.

### Pathological examination

Pathological diagnosis was carried out by pathologists at the screening units in accordance with uniform standards. Women with BI-RADS category 4 or higher underwent biopsy, and confirmed cases were staged according to the eighth-edition Breast Cancer Staging System published by the American Joint Committee on Cancer (AJCC).

Mammography screenings were carried out employing the Hologic Selenia Dimensions system (Hologic, Inc., Marlborough, MA, USA), a system renowned for its high-resolution imaging capabilities, thereby contributing to the accuracy of the diagnostic process.

### Statistical analysis

The distribution of malignant imaging findings across the BI-RADS categories from different imaging screening modalities was calculated to assess the prevalence of high classifications that would warrant clinical biopsy. The inter-modality agreement on BI-RADS category 4 or higher was assessed to evaluate diagnostic characteristics of each imaging method. The efficacy of breast cancer screening based on BI-RADS-guided referrals for pathological biopsy was evaluated using the diagnostic indicators of sensitivity, specificity, positive predictive value (PPV), negative predictive value (NPV), and the area under the receiver operating characteristic (ROC) curve. SAS 9.4 software was utilized for all statistical analyses, with a significance level set at α = 0.05.

## Results

### Breast cancer detection

Of 8996 women, 362 individuals were classified as BI-RADS category 4 or higher, and 117 biopsies were performed in the breast cancer screening program. Ultimately, 29 breast cancer cases were identified, with 4 cases in the 35–44 years group and 25 cases in the 45–64 years group.

### Imaging findings and BI-RADS classification

**Table 1** delineates the distribution of malignant imaging findings across the BI-RADS categories in HHUS, ABUS and MG. A minority of mass-related malignant signs were BI-RADS category 2 in both HHUS (48 women) and ABUS (44 women), while the majority were BI-RADS category 3 (318 for HHUS and 288 for ABUS) or BI-RADS category 4 or higher (438 for HHUS and 403 for ABUS). Notably, among mass-related malignant signs, indistinct margins and not parallel (taller than wide) orientation showed a high proportion of lesions being classified as BI-RADS category 4 or higher (81% and 72.7% in HHUS, 77.5% and 88.9% in ABUS, respectively). MG less frequently identified mass-related malignant signs, such as 31 for irregular morphology and 71 for indistinct margins, compared to HHUS (573 vs. 209) and ABUS (530 vs. 187). However, MG detected more calcification than HHUS (7 vs. 4). Among individuals with calcification, 100% were BI-RADS category 4 or higher for HHUS, whereas 85.7% for MG. Meanwhile, all eight individuals with architectural distortion detected by MG were BI-RADS category 3 or higher, of whom 62.5% were BI-RADS category 4 or higher. This diagnostic pattern is similar to that of the characteristics observed with calcifications, indicates that both architectural distortion and calcifications are strong indicators of malignancy for radiologists. Asymmetric density in MG was largely concentrated in BI-RADS category 3 (98.3%).

**Table 1:** The distribution of malignant imaging findings across different BI-RADS classification.

Method	Type	Malignant imaging findings	BI-RADS (n, %)			
			1	2	3	≥4
HHUS	Mass	Irregular Morphology	0(0)	43(7.5)	279(48.5)	251(44)
		Indistinct margins	0(0)	4(1.9)	34(16.1)	171(81.0)
		Not parallel (taller than wide) orientation	0(0)	1(4.5)	5(22.7)	16(72.7)
	Calcification	grouped or linear	0(0)	0(0)	0(0)	4(100)
	Associated Features	Architectural distortion	0(0)	0(0)	0(0)	0(0)
		Skin thickening	0(0)	3(100)	0(0)	0(0)
		Elastic imaging (hardness)	0(0)	0(0)	0(0)	0(0)
ABUS	Mass	Irregular Morphology	0(0)	42(7.9)	246(46.4)	242(45.7)
		Indistinct margins	0(0)	2(1.1)	40(21.4)	145(77.5)
		Not parallel (taller than wide) orientation	0(0)	0(0)	2(11.1)	16(88.9)
	Calcification	grouped or linear	–	–	–	–
	Associated Features	Architectural distortion	0(0)	0(0)	1(100)	0(0)
		Skin thickening	0(0)	0(0)	0(0)	0(0)
		Elastic imaging (hardness)	–	–	–	–
MG	Mass	Irregular Morphology	0(0)	0(0)	7(22.6)	24(77.4)
		Indistinct margins	0(0)	0(0)	42(59.2)	29 (40.8)
		High density	0(0)	0(0)	9(45)	11(55)
	Calcification	grouped or linear	0(0)	1(14.3)	0(0)	6(85.7)
	Associated Features	Architectural distortion	0(0)	0(0)	3(37.5)	5(62.5)
		Skin thickening	0(0)	0(0)	0(0)	0(0)
		Asymmetric density	0(0)	0(0)	289(98.3)	5(1.7)

HHUS, Handheld ultrasound; ABUS, Automated breast ultrasound; CI, Confidence Interva; BI-RADS, the Breast Imaging-Reporting and Data System.

### Agreement of BI-RADS classification across different imaging modalities

The agreement on BI-RADS classifications of category 4 or higher between HHUS and ABUS was 98.2%, with a Kappa coefficient of 0.726, indicative of a substantial agreement between the two imaging modalities. When stratified by age groups, the agreement for both 35–44 and 45–64 subgroups was found to be 98.2%. This suggests no significant variation in consistency between the two ultrasound techniques across different age groups (**Table 2**).

**Table 2:** The agreement of BI-RADS classification between HHUS and ABUS.

Age group	HHUS	ABUS		Total	Agreement (%)	Kappa (95%CI)
		Positive	Negative			
35–64 years	Positive	225(2.5)	84(0.9)	309	98.2	0.726 (0.6856,0.7664)
	Negative	78(0.9)	8607(99.1)	8685		
35–44 years	Positive	64(70.3)	27(29.7)	91	98.2	0.694 (0.6168,0.7715)
	Negative	27(0.9)	2918(99.1)	2945		
45–64 years	Positive	161(73.9)	57(26.1)	218	98.2	0.739 (0.6922,0.7867)
	Negative	51(0.9)	5689(99.1)	5740		

Positive indicates BI-RADS category 4 or higher. Negative indicates BI-RADS category 3 or lower.

HHUS, Handheld ultrasound; ABUS, Automated breast ultrasound; CI, Confidence Interval.

In contrast, the agreement between ultrasound and MG within the 45–64 age group was comparatively lower, with agreement rates of 96.1% for HHUS and MG, and 96.3% for ABUS and MG, respectively. This discrepancy primarily stemmed from a higher proportion of cases where ultrasound flagged as positive while MG flagged as negative (77.3% for HHUS and 77.6% for ABUS), versus fewer instances where ultrasound flagged as negative while MG flagged as positive (22.7% for HHUS and 22.4% for ABUS) (**Table 3**).

**Table 3:** The agreement of BI-RADS classification between ultrasound and MG in 45–64 years group.

Method	Result	MG		Total	Agreement (%)	Kappa (95%CI)
		Positive	Negative			
HHUS	Positive	35(16.3)	180(83.7)	215	96.1	0.2145 (0.1512,0.2778)
	Negative	53(0.9)	5670(99.1)	5723		
ABUS	Positive	39(18.7)	170(81.3)	209	96.3	0.2469 (0.1807,0.3132)
	Negative	49(0.9)	5682(99.1)	5731		

Positive indicates BI-RADS category 4 or higher. Negative indicates BI-RADS category 3 or lower.

HHUS, Handheld ultrasound; ABUS, Automated breast ultrasound; MG, Mammography; CI, Confidence Interval.

### Efficacy of BI-RADS classification in cancer screening

The efficacy of different image-based BI-RADS classification systems in breast cancer screening was evaluated across age groups. In the 35–44 age group, both HHUS and ABUS independently detected all 4 breast cancer cases of, exhibiting comparable performance metrics in sensitivity(75% vs 75%), specificity(98.55% vs 98.58%), PPV(6.52% vs 6.67%), and NPV(99.97% vs 99.97%). Among the 45–64 years group, ABUS detected all 25 confirmed breast cancer cases, HHUS detected 23 cancer cases, and MG only identified 20 cancer cases. The sensitivities were 100.0% for ABUS, 92.0% for HHUS, and 80.0% for MG, with specificities ranging from 98.6% to 99.7% (**Table 4**). Incorporating MG BI-RADS diagnoses into assessments of women with negative ultrasound findings (BI-RADS category 3 or lower) increased the sensitivity of HHUS from 92% to 100%, albeit with a minor decrease in specificity from 98.56% to 98.39%. A similar drop in specificity was observed for ABUS, from 98.66% to 98.55%. The evaluation of AUC yielded values of 0.9529 for HHUS, 0.9935 for ABUS, and 0.8983 for MG. The synergistic application of HHUS and MG, following the aforementioned combined assessment rules, significantly enhanced the AUC of HHUS, elevating it from 0.9529 to 0.9920. Notably, the AUC for ABUS combined with MG was slightly lower than when using ABUS alone.

**Table 4:** Performance metrics of the BI-RADS classification in cancer screening.

Method	35–44 years group					45–64 years group				
	Sensitivity	Specificity	PPV	NPV	AUC	Sensitivity	Specificity	PPV	NPV	AUC
HHUS	75.00 (30.06, 95.44)	98.55 (98.05, 98.92)	6.52 (2.24, 17.50)	99.97 (99.81, 99.99)	0.8678	92.00 (75.03, 97.78)	98.56 (98.21, 98.83)	21.70 (14.92, 30.46)	99.96 (99.87, 99.99)	0.9529
ABUS^*^	75.00 (30.06, 95.44)	98.58 (98.09, 98.95)	6.67 (2.30, 17.86)	99.97 (99.81, 99.99)	0.8679	100 (86.68, 100)	98.66 (98.33, 98.93)	24.51 (17.19, 33.68)	100 (99.93, 100)	0.9935
MG	–	–	–	–		80.00 (60.87, 91.14)	99.65 (99.46, 99.77)	50.00 (35.20, 64.80)	99.91 (99.80, 99.96)	0.8983
HHUS+MG^*^	–	–	–	–		100 (86.68, 100)	98.39 (98.04, 98.69)	21.37 (14.91, 29.64)	100 (99.93, 100)	0.9920
ABUS+MG^*^	–	–	–	–		100 (86.68, 100)	98.55 (98.21, 98.83)	23.15 (16.20, 31.94)	100 (99.93, 100)	0.9928

HHUS, Handheld ultrasound; ABUS, Automated breast ultrasound; MG, Mammography; PPV, Positive predictive value; NPV, Negative Predictive value; AUC, The area under the receiver operating characteristic (ROC) curve; HHUS+MG HHUS used for initial screening, HHUS-negative women undergo MG screening, ABUS+MG ABUS is used for initial screening, and MG screening is used for ABUS-negative women.

^*^Compared with mammography alone, significant differences were observed for AUC in the 45–64 years group.

## Discussion

This study found that almost all malignant signs identified by radiologists at primary healthcare hospitals were diagnosed as BI-RADS category 3 or higher, which alert clinical attention. Signs with high malignancy indications, such as not parallel (taller than wide) orientation and grouped calcifications, were categorized as higher, namely BI-RADS category 4 or higher, to prompt timely biopsies in clinical practice. Ultrasound is sensitive in detecting mass-related malignant signs, while MG is sensitive in detecting malignant calcifications and architectural distortion. The efficacy of BI-RADS classification used in breast cancer screening is promising in primary healthcare settings.

In this study, it was observed that irregularly shaped masses were similarly categorized as BI-RADS category 3 and category 4 or higher by HHUS and ABUS. However, in MG, these masses tended to be assigned higher BI-RADS levels. Considering the significantly lower detection of mass lesions by MG compared to ultrasound, it can be hypothesized that this difference may be related to the lower sensitivity of MG in detecting isodense lesions. Therefore, irregularly shaped masses visualized on MG often draw high attention from radiologists (31, 32). This study also indicated that in ultrasound examinations, features of indistinct mass margins were strongly associated with a categorization of BI-RADS category 4 or higher. This contrasted starkly with the findings of MG, which did not show a distinct preference for indistinct mass margins between BI-RADS category 3 and 4. This may be due to the fact that during the MG imaging process, the edges of many benign lesions become blurred due to the surrounding breast tissue, complicating the evaluation of these lesions (33, 34). Furthermore, regarding the orientation observed in the images, masses with a parallel (wider-than-tall) orientation are less likely to be malignant on ultrasound compared to those with a not parallel (taller-than-wide) orientation (35). In this study, masses with a not parallel (taller-than-wide) orientation were classified as BI-RADS category 4 or higher in 72.7% and 88.9% of HHUS and ABUS diagnoses, respectively.

In the context of breast cancer screening, calcifications are pivotal imaging findings, especially in MG examinations. Coarse or popcorn-like calcifications are often associated with benign lesions, while small and grouped, branching, or linear calcifications may indicate malignancy (15). The distribution and morphology of calcifications are more distinctly observable on MG than ultrasound, which is consistent with the findings of other studies. This is possibly because MG can more intuitively present calcifications (36).

The study demonstrated a high diagnostic consistency between HHUS and ABUS, indicating that ABUS can be effectively utilized for BI-RADS classification like HHUS. However, when comparing HHUS and ABUS to MG, the consistency was lower with 96.1% and 96.3% respectively. Discrepancies predominantly manifested as a higher proportion of cases (77.3% for HHUS and 77.6% for ABUS) where both HHUS and ABUS detected abnormalities while MG did not, as opposed to fewer instances (22.7% for HHUS and 22.4% for ABUS) where ultrasound flagged as negative while MG flagged as positive. The differences may be due to the varying abilities of ultrasound and MG to detect different types of malignancy-related pathological features observed in this study. Ultrasound may be more effective in identifying mass-related signs, while MG may be more excellent at detecting calcifications and architectural distortions. The reason why MG detects fewer mass-related signs compared to ultrasound may be related to the high-density glandular tissue that can obscure isodense lesions, while ultrasound is not affected by such tissue density (37). Our findings align with those of previous studies, which indicate that MG identifies fewer mass-related malignancies compared to ultrasound but detects a higher prevalence of malignancies associated with calcifications and architectural distortions (38–41), which highlights the complementary nature of ultrasound and MG in breast cancer screening.

This study also evaluated the performance of BI-RADS classification system in breast cancer screening, highlighting notable differences across age groups and screening methods. In the screening program for individuals aged 35-44, both HHUS and ABUS showed similar diagnostic performance, with sensitivities of 75% and specificities of 98.55% and 98.58%, respectively. For individuals aged 45-64, ABUS showed slightly higher sensitivity (100% vs 92%) and specificity (98.66% vs 98.56%). This aligns with a similar finding in a previous study that ABUS has statistically significant higher diagnostic accuracy than HHUS in detecting breast cancer (42). This difference may be attributed to ABUS providing volume and more comprehensive breast imaging, thereby enhancing the detection rate of lesions (43). When using MG as a supplementary examination to the ultrasound-negative women, the BI-RADS classification system can increase the sensitivity from 92% to 100% in the 45–64 age group, underscoring the benefits of a comprehensive screening approach. However, a minor decrease in specificity may result in an increase in false-positive results, imposing a psychological burden on patients and increasing the cost of follow-up examinations. Despite achieving a sensitivity of 100% when used alone, ABUS showed a decrease in AUC if using MG as a supplementary examination, suggesting that adding additional screening methods may not confer additional benefits to ABUS (44).

Nevertheless, this study has several limitations that should be considered when interpreting its results. Firstly, our study was conducted at a single center, which may limit the breadth of our findings. The practices of BI-RADS in primary healthcare in our study may not be fully representative of those found in other settings. Secondly, while our study evaluated the implementation of the BI-RADS classification system in primary healthcare hospitals, it did not involve a quality control assessment by highly experienced radiologists to directly compare and more accurately evaluate the standardization and compliance of BI-RADS application at these facilities.

## Conclusion

Radiologists at primary hospitals can effectively adhere to BI-RADS guidelines to provide clinical indications of malignant risks. The differences in BI-RADS classification diagnoses between ultrasound and MG reflect the characteristics of each imaging technique. Based on the BI-RADS findings, HHUS, ABUS, and MG have good efficacy in breast cancer screening. In conclusion, the application of BI-RADS is acceptable in primary healthcare hospitals.

## Data Availability

The original contributions presented in the study are included in the article/supplementary material. Further inquiries can be directed to the corresponding author.
